# Total suffering and will to live in persons with life-limiting diseases: Results from an Iberian multicenter study

**DOI:** 10.1017/S1478951525100278

**Published:** 2025-07-31

**Authors:** Miguel Julião, Bárbara Antunes, Carolina Simões, Maria Ana Sobral, André Oliveira, Carla Melo, Marco Mendonça, Milene Mendonça Lima, Giovanni Cerullo, Ana Carrancha, Duarte da Silva Soares, Daniela Artilheiro, Maria de Lurdes Pradinhos, Esperanza Begoña Garcia Navarro, Sonia García Navarro, Rosa Pérez Espina, Mathieu Bernard, Bianca Sakamoto Ribeiro Paiva, Marco Antonio de Oliveira, Harvey Max Chochinov, Eduardo Bruera

**Affiliations:** 1 Equipa Comunitária de Suporte em Cuidados Paliativos PalCo, ULS Amadora/Sintra, Amadora, Portugal; 2 Primary Care Unit - Department of Public Health and Primary care, University of Cambridge, Cambridge, UK; 3 Centro de Estudos e Investigação em Saúde da Universidade de Coimbra, Portugal; 4 Hospitalização Domiciliária Cuidados Paliativos CUF, Portugal; 5 Unidade de Cuidados Paliativos Agudos CUF Tejo, Lisboa, Portugal; 6 Unidade de Cuidados Paliativos do Hospital do Divino Espírito Santo de Ponta Delgada, EPER, Ponta Delgada, Açores, Portugal; 7 Equipa de Apoio Psicossocial do Hospital do Divino Espírito Santo de Ponta Delgada, EPER, Ponta Delgada, Açores, Portugal; 8 Unidade de Cuidados Paliativos e Equipa de Apoio Psicossocial do Hospital do Divino Espírito Santo de Ponta Delgada, EPER, Ponta Delgada, Açores, Portugal; 9 Equipa Comunitária de Suporte em Cuidados Paliativos da Unidade de Saúde da Ilha de São Miguel, Ponta Delgada, Açores, Portugal; 10 Serviço de Cuidados Paliativos, Unidade Hospitalar de Faro, Centro Hospitalar e Universitário do Algarve, Faro, Portugal; 11 Equipa Intrahospitalar de Suporte em Cuidados Paliativos, Unidade Hospitalar de Faro, Centro Hospitalar e Universitário do Algarve, Faro, Portugal; 12 Unidade Local de Saúde do Nordeste, Bragança, Portugal; 13 Equipa Intrahospitalar e Unidade de Cuidados Paliativos da Unidade Hospitalar de Macedo de Cavaleiros da Unidade Local de Saúde do Nordeste, Bragança, Portugal; 14 Department of Nursing, Director of Health, University of Huelva, Huelva, Spain; 15 Primary Care Unit - Department of Nursing, University of Huelva, Huelva, Spain; 16 Palliative Care Support Team, Juan Ramón Jiménez Hospital Area, Huelva, Spain; 17 Service of Palliative and Supportive Care, Lausanne University Hospital, Lausanne, Switzerland; 18 Barretos Cancer Hospital, Research Group on Palliative Care and Health-Related Quality of Life, Barretos, Brazil; 19 Department of Psychiatry, Research Institute of Oncology and Hematology, Cancer Care Manitoba, Manitoba, Canada; 20 Department of Palliative, Rehabilitation and Integrative Medicine, University of Texas MD Anderson Cancer Center, Houston, Texas

**Keywords:** Total suffering, will to live, multicenter study, palliative care, end of life

## Abstract

**Objectives:**

The concept of total suffering is widely recognized in palliative care (PC), encompassing a range of interconnected and complex factors that collectively shape the evolving and individualized experience of a patient’s illness journey. Studies on will to live (WtL) in terminally ill patients have demonstrated its variability over time and various factors that influence these changes.

**Methods:**

To objectively investigate the concept of total suffering and WtL; including their fluctuation over time and associations with sociodemographic, clinical, physical, and psychological symptoms in a sample of individuals with life-limiting conditions receiving PC. This multicenter Iberian study involved 3 centers in Portugal and 1 in Spain. A total of 107 individuals with life-limiting conditions consented to participate. To capture the dynamic and multifaceted components of total suffering, we had each participant completed the Edmonton Symptom Assessment Scale (ESAS) along an additional WtL visual analogue once daily over a 30-day period.

**Results:**

WtL demonstrated various patterns over time. While some patterns reflected relative stability, other demonstrated substantive fluctuation during the course of illness. Significant correlations were observed between WtL and all other ESAS items. Moderate positive correlations were found between WtL and total ESAS score and its physical and psychological sub-scores. Spearman’s correlation coefficients between all physical and psychosocial items on the ESAS were statistically significant across all 45 correlations performed, with only 5 showing moderate strength; the remaining correlations were weaker.

**Significance of results:**

Evidence-based understanding of WtL is critical to improving care for patients who experience suffering toward end-of-life and their families. Further research is needed to inform and refine interventions targeting total suffering.

## Introduction

Improving quality of life through the prevention and relief of suffering of persons with life-limiting diseases is one of the primary goals of palliative care (PC). Although there is a link between the expression of total suffering, diminished will to live (WtL), and uncontrolled symptoms, this relationship has not been widely researched (Bornet et al. 2021; Julião et al. 2020b).

Understanding suffering and its triggers contributes to early detection and prevention, ultimately leading to more humane and respectful approaches toward patients, families, and caregivers (Dees et al. 2011; Krikorian and Limonero 2012). Total suffering is defined as a state of severe physical and emotional distress caused by events that threaten the integrity of an individual as a complex and social entity (Abraham et al. 2006; Cassell 1982).

It is the one of the most debilitating conditions experienced by persons with life-limiting diseases. However, the absence of a universally agreed-upon definition of holistic suffering and its complex, multidimensional taxonomy remains a barrier to progress (Dees et al. 2011).

WtL is described as “the psychological expression of one’s commitment to life and the desire to continue living, encompassing both instinctual and cognitive-emotional components” (Mosby´s Medical Dictionary, 2008; Carmel et al. 2007). WtL is a psychobiological phenomenon that encompasses both rational and irrational components (Hudson et al. 2006). Research involving patients with advanced cancer has revealed significant negative correlations between WtL, physical symptoms, anxiety, and positive correlations with overall well-being (Hudson et al. 2006).

Tataryn and Chochinov (2002) examined the trajectory of WtL in persons with life-limiting diseases, concluding that WtL is highly variable during the terminal phase of illness. They reported WtL strongly correlates with anxiety, depression, and physical symptom distress, with these fluctuations intensifying as death approaches. Their findings suggest that patients with persistently low WtL often experience heightened symptomatology and may lack adequate social support. This underscores that WtL is complex, multidimensional, and influenced by physical and psychosocial sequela of disease.

Two Portuguese studies investigated themes related to the desire for death and WtL, focusing on small patient samples or case reports (Julião et al. 2020a, 2020b), utilized quantitative measures to explore the concept of total suffering and WtL. They concluded that it represents a complex and interconnected network of symptoms that can’t be understood or addressed separately. These efforts emphasize the need for further multicenter studies in PC exploring WtL, including a longitudinal perspective.

To shed further light on total suffering, this study aims to determine (1) the correlations between WtL and all physical and psychological items on the Edmonton Symptom Assessment Scale (ESAS) in persons with life-limiting diseases; (2) the association between WtL and various sociodemographic and disease-related factors; (3) the relationship between WtL and patient-reported ESAS physical and psychological subscales and total score; and (4) the trajectory and patterns of WtL fluctuation over time.

## Methods

### Design, participants, and study sites

This was a prospective, observational, multicenter study. A convenience sample of 107 persons with life-limiting diseases was recruited from 4 PC centers: 3 in Portugal (Macedo de Cavaleiros, Algarve, and Açores) and 1 in Spain (Huelva). All centers provided tertiary PC programs, encompassing home-based, outpatient, and inpatient services. All participants were inpatients, who were consecutively recruited over 6 months from January to June 2022.

Study participants had to be 18 years of age or older and diagnosed with a life-threatening disease with a prognosis of at least 3 months based on clinical assessment. Participants could not show the evidence of dementia or delirium (as determined by chart review or clinical consensus) and required a Mini-Mental State Examination score of 20 or higher. Additionally, participants had to be able to read and speak their respective mother tongue, provide written informed consent, and commit to completing the ESAS at the same time daily for 30-days.

### Measures

The ESAS is the most widely used self-report assessment tool in PC, which is employed for daily clinical evaluations across various patient settings (Bernardo 2005; Carvajal et al. 2013). It assesses 9 core symptoms including pain, depression, anxiety, shortness of breath, appetite, nausea, tiredness, drowsiness, and well-being; for the purposes of this study, we added an additional item addressing WtL. Each ESAS item is scored on a scale from 0 (no symptom burden) to 10 (extreme symptom burden). The total ESAS score is calculated by summing the scores of all 9 core symptoms (excluding WtL), with a range of 0 (no symptom burden) to 90 (worst possible symptom burden). The ESAS physical sub-score includes pain, shortness of breath, appetite, nausea, tiredness, drowsiness, and well-being items, with scores ranging from 0 (no symptom burden) to 70 (worst possible symptom burden). The ESAS psychological sub-score includes the depression and anxiety items, with scores ranging from 0 (no symptom burden) to 20 (worst possible symptom burden). The WtL item was scored on a scale from 0 (best possible WtL) to 10 (worse possible WtL/no WtL). Given the variability in how the concept of WtL might be understood and its potential for subjective interpretation, participants received a standardized explanation of the construct.

### Sociodemographic and clinical information

Sociodemographic and clinical data were collected directly from participants, their proxies, or medical records. This information included age, gender, marital status, social support, work status, and education level; primary diagnosis, time since diagnosis, performance status (Palliative Performance Scale [PPS]); current palliative treatments, previous PC follow-up, and prognostic awareness.

### Procedure

A research team member at each site screened newly admitted patients for eligibility. Eligible patients were informed about the study’s objectives and protocol details. Participants provided written informed consent before data collection commenced.

Each participant was asked to complete the ESAS/WtL once daily, at the same time every day, for 30 consecutive days. Cognitive status was monitored daily by a healthcare professional. In the event of clinical deterioration, data collection for that patient was stopped. Throughout the study, participants continued to receive regular, multi-professional PC, which included ongoing assessment and responding to physical, existential, and psychosocial needs.

### Data analysis

The ESAS, demographic and clinical characteristics were described using measures of central tendency and dispersion for continuous variables and proportions for categorical data. We applied generalized estimating equations using independent correlation and maximum likelihood estimation to explore the variables’ effects on WtL and the trajectory of WtL over time. Each participant identification number was entered into the statistical model as a random effect. The 30 evaluation times and WtL were considered within-subject variable. We further explored the trajectory of WtL by applying a least-squares linear regression procedure, yielding the slope and the intercept for each participant. Slope parameters are unstandardized betas and interpretable as millimeters of change in WtL-score per 30-day observation period. For this analysis, we used the GLM function (lme4 package) by R software (version 4.4.0).

Correlations between ESAS (individual items and subscales) were calculated using the Spearman rank correlation coefficient (*r*_s_), with Bonferroni correction (significance level divided by the number of tests) for multiple testing. Correlation coefficients are classified as very high (*r*_s_ = 0.90–1.00), high (*r*_s_ = 0.70–0.90), moderate (*r*_s_ = 0.50–0.70), and low (*r*_s_ 0.30–0.50) (Hinkle et al. 2003). This matrix was assembled to illustrate the complexities of total suffering. Internal consistency measures of reliability were calculated using Cronbach’s alpha coefficient and the respective confidence intervals were calculated using the procedure of Feldt et al. (1987). Statistical significance was 0.05 and Statistical Package for the Social Sciences (SPSS) software (version 21; SPSS, Inc., Chicago, IL) was used to perform the analysis.

## Results

### Participants

A total of 155 patients meeting eligibility criteria were assessed for interest and willingness to participate. Of those approached, 48 declined: 35 due to clinical deterioration, and 13 for unspecified reasons. The remaining 107 patients consented to take part: 77 from Portugal (Macedo de Cavaleiros, *n* = 18; Açores, *n* = 36; Algarve, *n* = 23), and 30 from Spain (Huelva, *n* = 30). No participants were lost due to clinical deterioration. Fifty-three percent of the patients were male and the majority were married (55.4%). The mean age was 65.7 years (range, 21−93). All participants were inpatients, and the most common diagnosis was cancer (90.6%). Adherence to ESAS self-reporting was 69.3%. There was no significant difference in WtL between the participating centers (*p* < 0.547). Total WtL mean was 2.4 (standard deviation: 2.4; [min = 0, max = 10]) with a median of 1.9. Total Cronbach alpha coefficient for ESAS was 0.85 (CI 0.84–0.86). See Table 1 for participant characteristics.

**Table 1.** Participants’ characteristics (*N* = 107)

	**Total** (N = 107)
**Age**, mean (SD), median [range], years	65.7 (15.3), 66 [21–93]
Gender, *n* (%)	
Male	57 (53.3)
**Marital status**, *n* (%)	
Married/living together	57 (55.4)
Single	10 (9.7)
Widowed	15 (14.6)
Divorced	21 (20.4)
**Educational level**, *n* (%)	
None	14 (13.6)
Elementary	59 (57.3)
High school	16 (15.5)
University	11 (10,7)
Master	1 (1.0)
Doctorate	2 (1.9)
**Family support**, *n* (%)	
Yes	100 (95.2)
No	5 (4.8)
**Diagnosis**, *n* (%)	
Cancer	97 (90.7)
Advanced organ disease	4 (3.7)
Neurodegenerative	2 (1.9)
Other	4 (3.7)
**Time since diagnosis**, *n* (SD), median [range] years	3.4 (5.9), 2 [0−38]
**Prior PC follow-up**, *n* (%)	
Yes	73 (68.9)
**Initial PPS**, *n* (%) median [range]	60.8 (14.1), 60 (30−90)
**Final PPS**, *n* (%) median [range]	49.9 (18,1), 50 (0−80)
**Will to live**, mean (SD), [range]	2.3 (2.7), [0−10]
**ESAS total**, mean (SD), [range]	25.0 (15.1), 22[0−87]
**ESAS psychological**, mean (SD), [range]	5.7 (4.7), [0−20]
**ESAS physical**, mean (SD), [range]	19.3 (11.8), [0−67]

ESAS = Edmonton Symptom Assessment Scale, PC = palliative Care, PPS = Palliative Performance Scale, SD = standard deviation.

### Correlation between WtL and all physical and psychosocial items on the ESAS

We used Spearman’s correlation coefficients to assess the relationships between all ESAS items (physical, psychological, and WtL). Table 2 shows low to moderate correlations across all 45 correlations between ESAS items after correcting for multiple testing.

**Table 2.** Spearman’s correlations between all ESAS symptoms

	**ESAS will to live**	**ESAS pain**	**ESAS depression**	**ESAS shortness of breath**	**ESAS appetite**	**ESAS nausea**	**ESAS tiredness**	**ESAS drowsiness**	**ESAS anxiety**	**ESAS well-being**
**ESAS will to live** *r_s p_*_value_										
**ESAS pain** *r_s p_*_value_	0.296 **<0.0001**									
**ESAS depression** *r_s p_*_value_	0.474 **<0.0001**	0.260 **<0.0001**								
**ESAS shortness of breath** *r_s p_*_value_	0.201 **<0.0001**	0.275 **<0.0001**	0.170 **<0.0001**							
**ESAS appetite** *r_s p_*_value_	0.415 **<0.0001**	0.292 **<0.0001**	0.333 **<0.0001**	0.147 **<0.0001**						
**ESAS nausea** *r_sp_*_value_	0.260 **<0.0001**	0.323 **<0.0001**	0.292 **<0.0001**	0.272 **<0.0001**	0.313 **<0.0001**					
**ESAS tiredness** *r_s p_*_value_	0.402 **<0.0001**	0.375 **<0.0001**	0.388 **<0.0001**	0.250 **<0.0001**	0.463 **<0.0001**	0.255 **<0.0001**				
**ESAS drowsiness** *r_s p_*_value_	0.206 **<0.0001**	0.270 **<0.0001**	0.302 **<0.0001**	0.213 **<0.0001**	0.245 **<0.0001**	0.285 **<0.0001**	0.296 **<0.0001**			
**ESAS anxiety** *r_s p_*_value_	0.459 **<0.0001**	0.322 **<0.0001**	**0.658** **<0.0001**	0.170 **<0.0001**	0.323 **<0.0001**	0.306 **<0.0001**	0.458 **<0.0001**	0.168 **<0.0001**		
**ESAS well-being** *r_s p_*_value_	**0.530** **<0.0001**	0.427 **<0.0001**	0.473 **<0.0001**	0.250 **<0.0001**	**0.574** **<0.0001**	0.305 **<0.0001**	**0.550** **<0.0001**	0.244 **<0.0001**	**0.537** **<0.0001**	

Moderate correlations are marked in bold.

ESAS = Edmonton Symptom Assessment Scale, *r_s_* = Spearman’s correlation coefficient.

### Association between WtL and sociodemographic and disease-related factors

In all adjusted models, a significant effect (*p* < 0.001) of the observation period on the mean WtL was estimated, indicating significant changes in mean WtL over time. The mean WtL value was higher among individuals aged 20–29 years (mean = 3.0; SD = 3.3) and 30–39 years (mean = 3.3; SD = 2.6), with a significant effect (*p* < 0.001) of the interaction between time and age group, highlighting the fluctuating nature of WtL in these groups (see Table 3).

**Table 3.** Associations between WtL and sociodemographic and disease-related factors

		**WtL mean (SD)**^a^	**Effect**^b^	*p* Value
**Age**	20–29	3.0 (3.3)	Moment	**<0.001**
	30–39	3.3 (2.6)	Age	0.246
	40–49	1.7 (2.7)	Moment × Age	**<0.001**
	50–59	2.3 (3.1)		
	60–69	2.4 (2.9)		
	70–79	2.1 (2.5)		
	>80	2.3 (2.3)		
**Gender**	Male	2.5 (2.7)	Moment	**0.001**
	Female	2.1 (2.7)	Gender	0.397
			Moment × Gender	**0.002**
**Marital status**	Married	2.2 (3.0)	Moment	**<0.001**
	Divorced/separated	2.5 (2.6)	Marital status	0.846
	Widowed	2.6 (2.5)	Moment × Marital Status	**<0.001**
	Living together	2.0 (2.0)		
	Single	2.1 (2.6)		
**Education**	No education	2.1 (2.3)	Moment	>0.99
	Elementary	1.9 (2.4)	Education	>0.99
	High school	2.6 (3.2)	Moment × Education	>0.99
	University	2.4 (2.5)		
	Master	1.9 (0.7)		
	PhD	2.9 (1.2)		
**Profession**	Primary sector	1.8 (2.3)	Moment	>0.99
	Secondary sector	3.7 (4.1)	Education	>0.99
	Third sector	2.4 (2.6)	Moment × Profession	>0.99
	Unemployed	2.6 (2.9)		
	Retired	2.2 (2.5)		
**Family**	No	1.0 (1.5)	Moment	**<0.001**
	Yes	2.3 (2.7)	Familiar support	**0.001**
			Moment × familiar support	**0.001**
**Diagnosis**	Cancer	2.9 (3.1)	Moment	**<0.001**
	Advanced organ disease	2.0 (2.0)	Diagnosis	**<0.001**
	Neurodegenerative	6.8 (3.4)	Moment × Diagnosis	**<0.001**
	Metasized cancer	1.9 (2.3)		
	Other	0.9 (1)		
**Time since diagnosis**	<1 year	1.9 (2.7)	Moment	**<0.001**
	≥1 e < 5 years	2.7 (2.8)	Time since diagnosis	**<0.001**
	≥5 e < 10 years	2.3 (2.4)	Moment × Time since diagnosis	**<0.001**
	≥10 e < 15 years	2.9 (2.6)		
	≥15 years	0.5 (0.8)		
**Palliative care follow-up**	No	2.7 (3.0)	Moment	**<0.001**
	Yes	2.2 (2.6)	Palliative care follow-up	0.277
			Moment × Palliative care follow-up	**<0.001**
**Initial PPS**	<50%	2.6 (3.2)	Moment	**<0.001**
	≥50%	2.3 (2.7)	Initial PPS	0.709
			Moment × Initial PPS	**<0.001**

PPS = Palliative Performance Scale.

a For the entire monitoring period.

b Effect obtained by application of generalized estimating equations.

Female participants exhibited a lower mean WtL value (mean = 2.1; SD = 2.7). We found a significant interaction (*p* = 0.002) between time and gender. This means that although WtL was similar for men and women in early observations, men tended to have worse WtL than women and later improved.

Data for divorced/separated participants (mean = 2.5; SD = 2.6) and widowed participants (mean = 2.6; SD = 2.5) showed higher mean WtL values, with married or cohabiting participants exhibited lower WtL (mean = 2.2; SD = 3.0) and (mean = 2.0; SD = 2.0), respectively. The significant interaction (*p* < 0.001) between time and marital status highlights the change in mean WtL of married participants over time, with a lower mean WtL until the 7th day and an increasing (worsening WtL) trend after the 8th day.

Educational level and profession were not found to be associated with WtL values (*p* > 0.99). We observed that the participants whose education level was PhD and High School reported the highest mean WtL values (mean = 2.9; SD = 1.2 and mean = 2.6; SD = 3.2, respectively). Participants whose profession was in the secondary sector reported the highest mean WtL values (mean = 3.7; SD = 4.1).

WtL values were lower (i.e., stronger WtL) for participants with prior PC follow-up (mean = 2.2; SD = 2.2) relative to those not being followed (mean 2.7; SD = 3.0). The significant interaction (*p* < 0.001) was observed mainly among patients without PC support, reflecting fluctuations in mean WtL over time in this group.

Participants without family support exhibited lower mean WtL values (mean = 1.0; SD = 1.5; *p* = 0.001). The significant interaction (*p* < 0.001) was mainly observed among patients without family support, due to the large fluctuations in mean WtL over time in this group.

The time since diagnosis also had a significant effect on mean WtL values, with participants diagnosed for more than 15 years showing lower mean WtL values compared to other time intervals. The interaction (*p* < 0.001) was due to large fluctuations in mean WtL among participants diagnosed 10–15 years ago.

### Association between WtL and patient-reported ESAS total score and ESAS subscales scores (psychological and physical)

**Figure 1.**
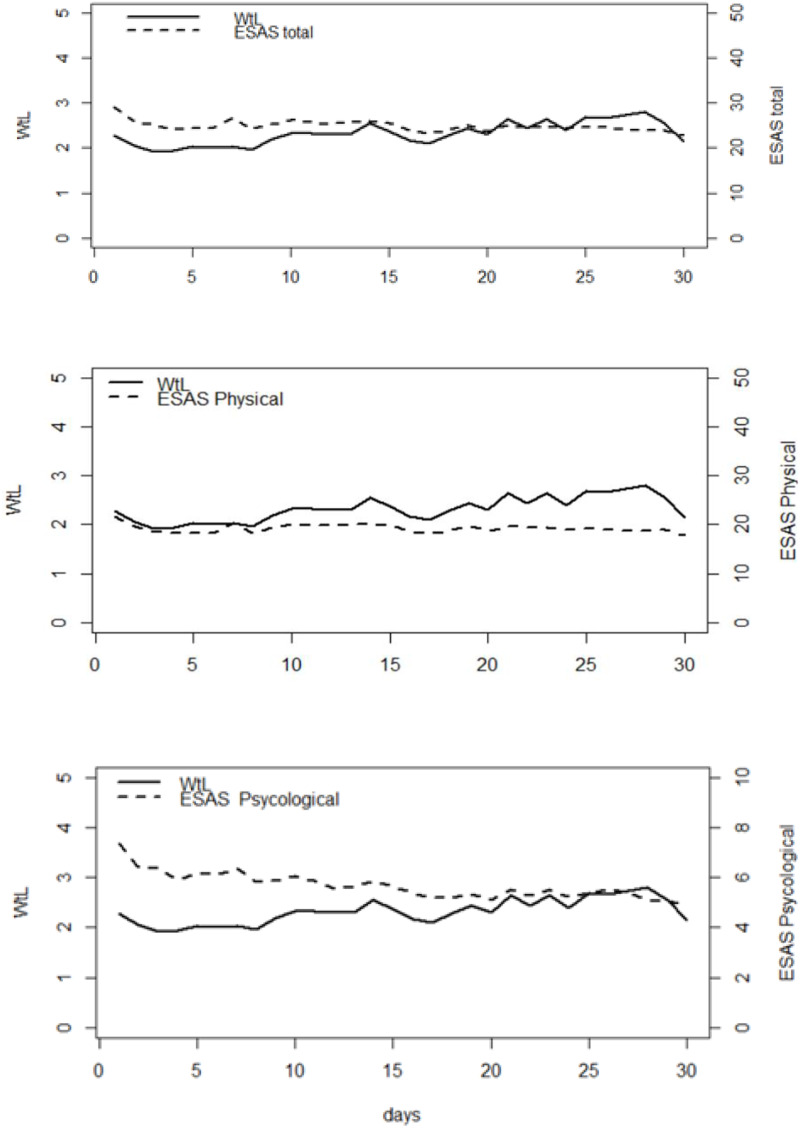
Time plots of the daily means WtL and the ESAS total score, ESAS physical and psychological scores.

Throughout the observation period, moderate positive correlations were found between WtL and ESAS total score (*r*_s_ = 0.565; *p* < 0.001); WtL and ESAS physical sub-score (*r*_s_ = 0.520; *p* < 0.001); and WtL and ESAS psychological sub-score (*r*_s_ = 0.517; *p* < 0.001) (Fig. 1).

### Regression modeling of WtL trajectories

**Figure 2.**
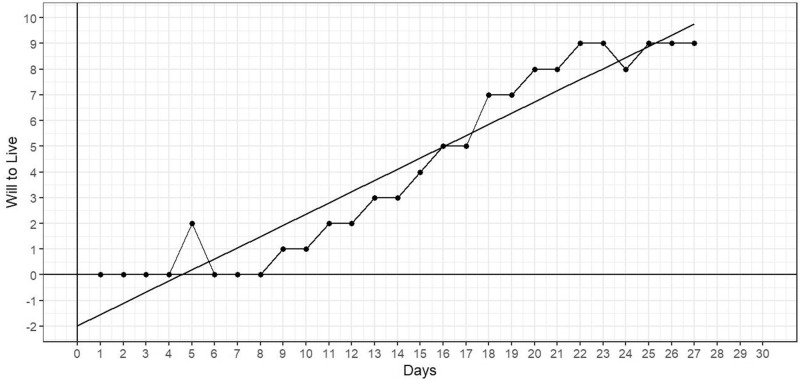
Will-to-live ratings over 30-day observation period for a 38-year-old woman with gastric cancer.

Using least-squares linear regression methodology, we determined the best-fit or trend line for WtL data provided over the 30-day observation period. By way of example, the trendline for data provided by this 38-year-old woman with gastric cancer yielded a slope of 0.44 (with an intercept of −1.98) (Fig. 2).

**Figure 3.**
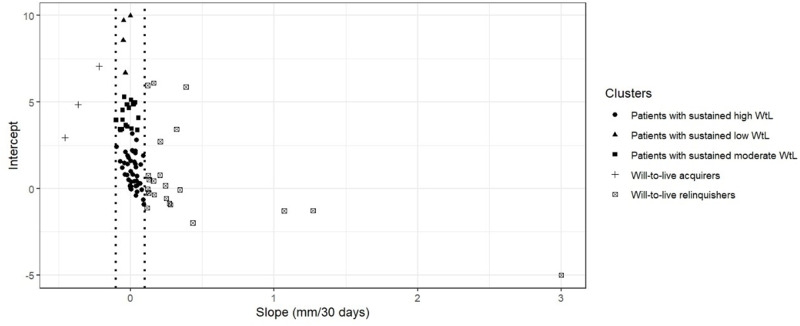
Least-squares linear regression parameters for 107 palliative care patients and WtL trajectory clusters.

We generated a point-plot of the slope and intercept parameters for all study participants (see Fig. 3). Points positioned to the right of zero on the horizontal axis (positive slope) indicate patients whose WtL diminishes over time; those to the left of zero (negative slope) represent patients who develop a stronger WtL over time. Points at or near zero indicate little to no change in WtL over time (see Fig. 3). Our analysis revealed 5 intercept-slope clusters:

1. Sustained high WtL (*n* = 56; 54%): These patients have consistently high WtL (low WtL scores). Their intercept-slope points cluster near 0 (±0.1) on the slope axis, with intercepts falling within the lower third of the WtL intercept axis.
2. Sustained low WtL (*n* = 4; 4%): These patients have consistently low WtL (high WtL scores). Their intercept-slope points cluster near 0 (±0.1) on the slope axis, with intercepts falling within the upper third of the will-to-live intercept axis.
3. Sustained moderate WtL (*n* = 18; 17%): These patients have consistently moderate WtL (medium WtL scores). Their intercept-slope points cluster near 0 (±0.1) on the slope axis, with intercepts falling within the middle third of the WtL intercept axis.
4. WtL relinquishers (*n* = 23; 22%): These patients demonstrate net relinquishment of WtL. For this cluster, least-squares linear regression yields positive slopes (>0.1), meaning higher WtL scores (poorer WtL) emerge over time.
5. WtL acquirers (*n* = 3; 3%): These patients demonstrate net acquisition of WtL. For this cluster, least-squares linear regression yields negative slopes (<0.1), meaning lower WtL scores (greater WtL) emerge over time.

## Discussion

To the best of our knowledge, this is the first multicenter study conducted in the Iberian region that used ESAS and WtL data to examine total suffer within a large sample of persons with life-limiting diseases cared for in PC.

### Correlation between WtL and physical and psychosocial items

The intricate web of psychological, social, and physical symptoms observed in this study aligns closely with the seminal work of Dame Cicely Saunders and others on the concept of “total pain/suffering” in PC (Saunders 1964; Clark 1999; Cassell 1982; Chochinov et al. 2005a; Wood 2022). Dame Saunders emphasized the interconnectedness between physical, psychological, social, and spiritual dimensions of suffering, asserting these must be addressed holistically. Our study affirmed WtL was significantly influenced by physical symptoms and emotional symptoms, social support, and relational dynamics. The interplay between these factors underscores the need for a comprehensive, multidisciplinary approach to PC, attending to all sources suffering within the social context of where patients live. Care that embraces and attends to this complexity has the capacity to bolster resilience and hope.

### Association between WtL and sociodemographic and disease-related factors

Younger participants demonstrated poorer WtL, consistent with other research examining age toward end-of-life (Gagliese et al. 2009; Khan et al. 2010). Youth is characterized by aspirations and ambitions. Having limited time and opportunity to achieve significant personal, family, and professional goals weighs heavily on patients anticipating an untimely death. This may lead to frustration, despair, existential distress, contributing to loss of WtL. The disparity between prior expectations and limited time can intensify feelings of grief for a “lost future,” undermining WtL. Aging provides opportunities to fulfil many life goals, suggesting older patients are better situated to reconcile their circumstances relative to those dying too young (Khan et al. 2010).

These insights underscore the importance of psychological and existential supports tailored for younger individuals in PC. Interventions focused on goal adaptation, legacy creation, and meaning making could be crucial in mitigating distress and fostering a sense of purpose, potentially improving WtL despite the constraints of their illness (Breitbart et al. 2015; Chochinov et al. 2005b, 2011).

Female participants in our study demonstrated stronger WtL than male participants. While WtL for women was reported to be lower (Carmel 2001, 2012), those findings were based on a cohort of elderly individuals not imminently facing death. Studies taking place more proximate to death reveal interesting gender differences. For example, Busquet-Duran et al. (2021) reported that women over 80 receiving home PC demonstrated less complexity with symptom management (41.7% vs. 51,1%; *p* = 0.011), emotional distress (24.5% vs. 32.8%; *p* = 0.015), spiritual distress (16.4% vs. 26.4%; *p* = 0.001), and socio-familial distress (62.7% vs. 70.1%; *p* = 0.036). Another PC study (Chochinov et al. 2006) found women were more likely to identify change in appearance, the ability to think clearly and a meaningful spiritual life as being dignity-related issues. Even integrating prognostic information seems be influence by gender, with women more likely to recognize their illness as incurable, know their cancer is at an advanced stage, and report having had discussions of life expectancy with their oncologist (Fletcher et al. 2013). It would appear, whether referring to WtL, spiritual distress, factors that infringe on dignity, or prognostic awareness, psycho-spiritual gender-based wiring helps shape end-of-life experience.

Participants who were divorced, widowed, or living alone tended to show poorer WtL. This suggests an interplay between psychological factors and social circumstances associated with isolation and loss. Absence of relational responsibilities may amplify loneliness, disconnection, and lack of support, further eroding WtL in the face of severe illness. Lacking close companions or familial network can limited access to emotional support or practical caregiving assistance, which can intensify the psychological burden of terminal illness. This isolation may impede access to shared decision-making or collaborative coping strategies, which are often facilitated by family or partners. Consequently, divorced, widowed, and single participants may experience a degree of existential distress undermining their WtL. Conversely, those with family support exhibited stronger WtL, perhaps reflecting emotional and practical benefits that enhance their sense of security, connection, and overall well-being.

Participants with prior PC experience demonstrated higher WtL, perhaps because of the pre-emptive nature of PC that prevents or effectively responds to suffering. Participants with higher education had stronger WtL, which could relate to better socioeconomic conditions and greater access to resources. Higher education often correlates with greater financial stability and better access to healthcare, both of which can impact patients’ sense of agency and outlook (Feinstein 1993). Those with higher education might be more likely to engage in reflective thinking, seeking meaning and purpose in their lives, even as they confront serious illness. Individuals with less education might experience more significant socioeconomic hardships, which may exacerbate isolation or despair.

The relationship between neurodegenerative diseases and low WtL is multifaceted, reflecting the impact of physical symptoms as well as psychosocial sequelae of progressive illness. Patients with neurodegenerative diseases, such amyotrophic lateral sclerosis, face significant emotional and physical distress as they become more aware of their cognitive decline, feeling a burden, and its impact on quality of life (Fisher et al. 2019; Nishiyama et al. 2024). As these diseases advance, patients may lose the ability to engage in meaningful social interactions and maintain independence, leading to isolation and loneliness. Furthermore, the burden of caregiving can create additional stress, as families and loved ones struggle to cope with the increasing needs of their loved ones. These factors can diminish sense of self-worth and purpose, undermining WtL in individuals with neurodegenerative diseases.

### Association between WtL and ESAS subscales, and total score

We found moderate associations between WtL, and physical and psychological dimensions of the ESAS. This suggests that WtL is multidimensional and influenced by various facets of the patient’s experience. It also affirms that the antecedents of suffering are multidetermined (Weisman and Worden 1976; Chochinov, 2005a). Strong WtL tends to align with resilience to stress, anxiety, and depression. A positive attitude, even in the face of severe adversity, correlates with lower psychological distress; heightened purpose elicits more engagement in adaptive coping strategies, mitigating mental health disorders such as depression. By contrast, diminished WtL is linked with hopelessness, leading to increased vulnerability to suicidal ideation and depression. WtL may also mediate physical dimensions of chronic illness, with strong WtL associated with longer survival (Carmel et al. 2007).

### Trajectory of WtL over time, exploring patterns of fluctuation

Consistent with previous studies (Chochinov HM, Tataryn D, Dudgeon D, Clinch J, 1999; Julião et al. 2020b; Tataryn and Chochinov 2002), our findings affirm the various trajectories of WtL that emerge across individuals facing imminent death. We identified 5 unique patterns of WtL, with the most common being “sustained high WtL” (54%). This highlights the resiliency of WtL, even for patients approaching death. Two other stable WtL patterns emerged, including “sustained moderate WtL” (17%) and “sustained low WtL” (4%). The latter finding affirms that it is not common for patients at end-of-life to consistently maintain low WtL. The 2 most dynamic patterns we identified include, “WtL relinquishers” (22%) and “WtL acquires” (3%). Understanding these patterns, and research exploring the physical, psychosocial, and spiritual complexities embedded within these patterns, will enable more response and effective PC. It also provides an empirical basis for unravelling the complexities of an expressed wish to die. Responding to requests for hastened death demands that clinicians be acutely aware of factors assaulting WtL, the variable patterns WtL manifests nearing death, and patient-centered interventions that might restore hope or purpose.

## Limitations

This study has several limitations that should be acknowledged. First, the sample was limited to hospitalized patients, who might be subject to a higher symptom burden. This may have influenced the assessments of both the WtL and the ESAS. Second, the patient population was predominantly composed of individuals with oncological conditions. Lastly, all participants were receiving care from a specialized PC team, which may have ensured better symptom control and, in turn, influenced the WtL scores in a more favorable direction. These factors should be considered when interpreting the findings and addressed in future research to enhance generalizability.

## Conclusion

This study affirms the complex nature of suffering and WtL in individuals approaching death. It establishes the multiplicity of influences on WtL, including demographic, psychosocial, and disease-specific factors. Clinicians must understand this complexity to help patients navigate the proclivities of shifting WtL. Our evidence underscores the need for approaches that are holistic, mindful of distinctive patterns WtL can follow. Our study also demonstrates the benefits of integrating patient centered outcome measures in daily practice. Not only are they easily administered but also valuable in unravelling – and hence addressing – the myriad influences on suffering for patients nearing death.
